# Supplementary material for: Integrated Analysis of Gut Microbiome and Adipose Transcriptome Reveals Beneficial Effects of Resistant Dextrin from Wheat Starch on Insulin Resistance in Kunming Mice
Source: Biomolecules. 2024 Feb 4;14(2):186. doi: 10.3390/biom14020186 (PMC10886926; doi:10.3390/biom14020186)
Supplement: Supplementary file 1 [file biomolecules-14-00186-s001.zip › biomolecules-2780435-supplementary.pdf]

Supplementary Figures

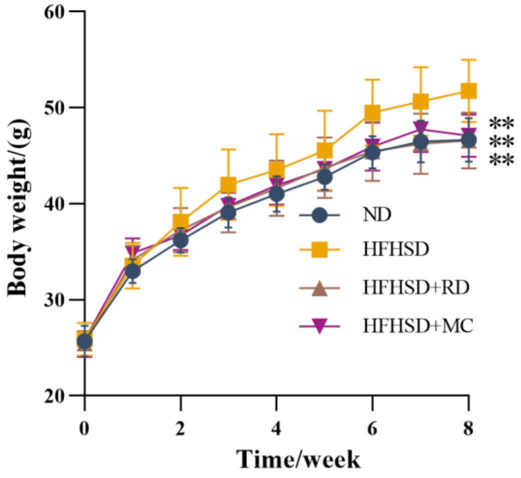

Figure S1: Change in body weight of mice in each group.

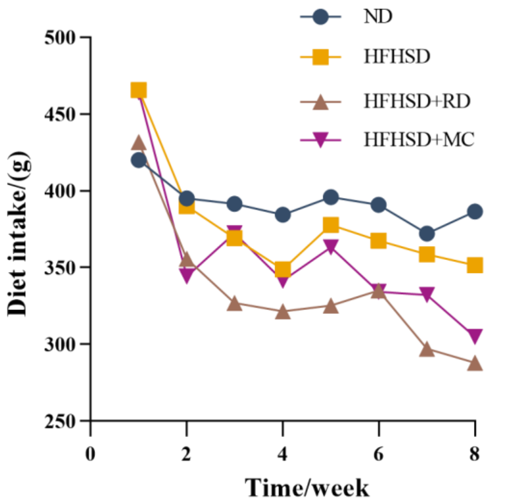

Figure S2: Dietary changes in four groups of mice

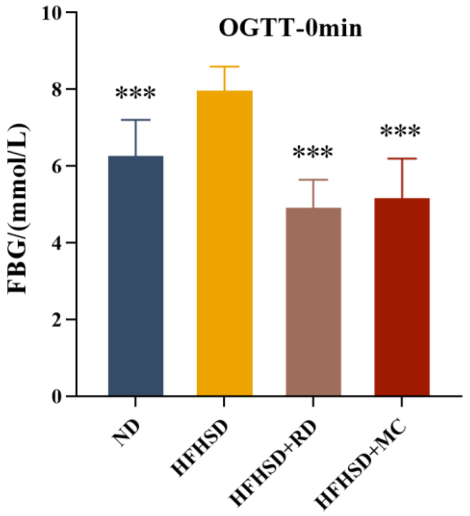

Figure S3: Fasting blood glucose in each group of mice during oral glucose tolerance test (0 min).

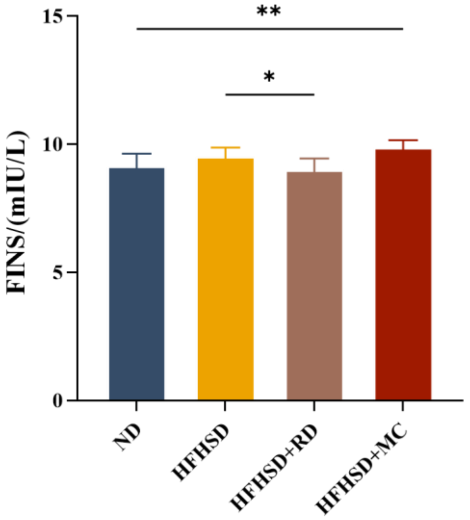

Figure S4: Serum fasting insulin levels in mice at week 8.

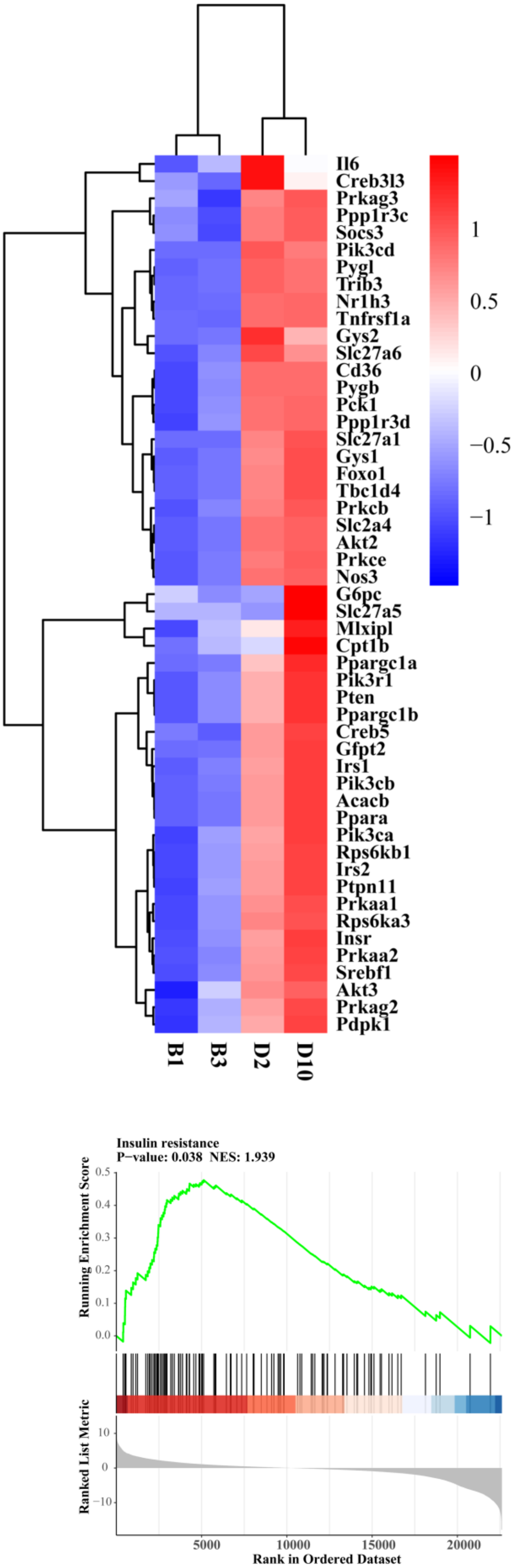

Figure S5: Gene expression changes in insulin resistance signaling pathway (GSEA).

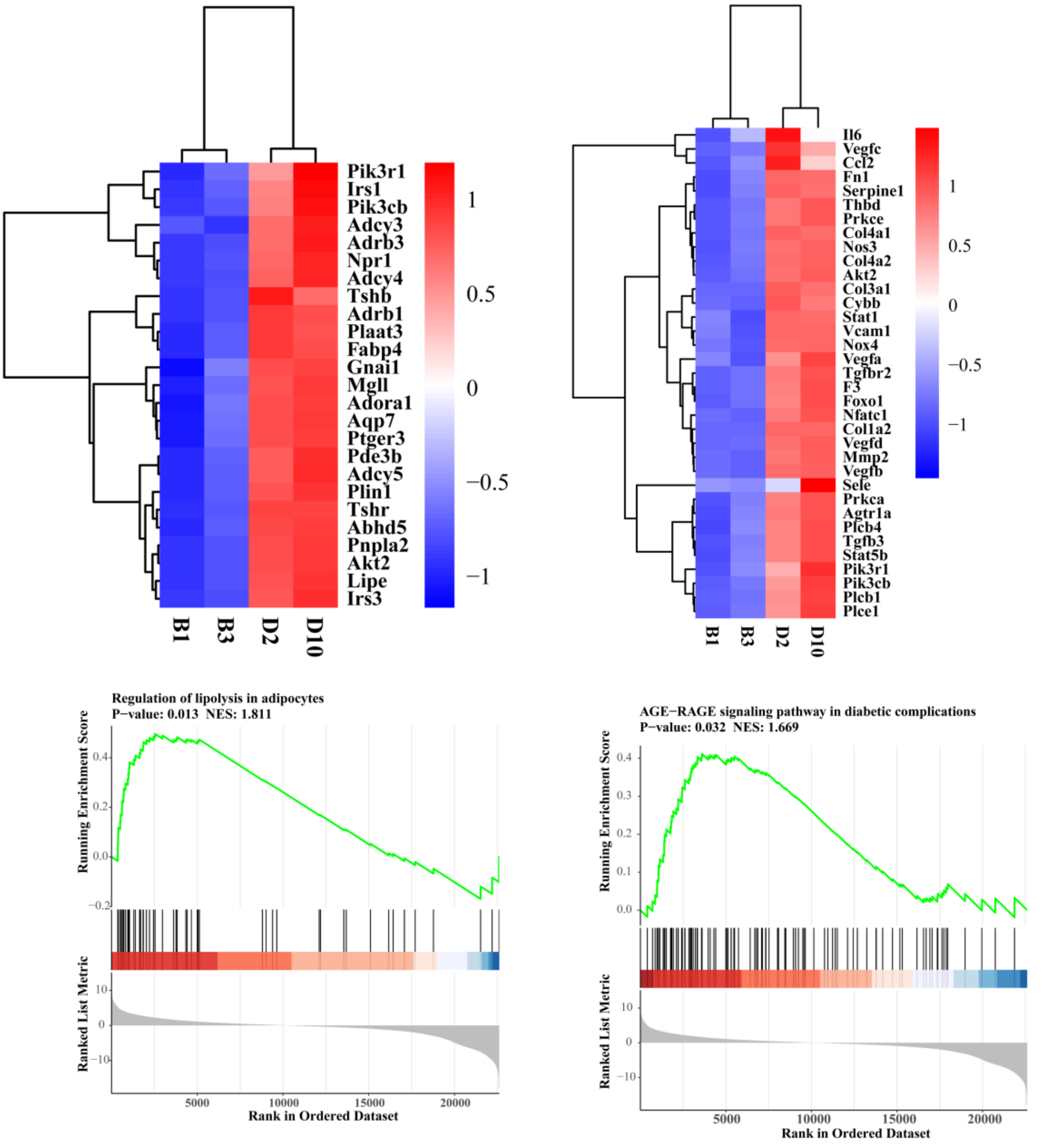

Figure S6: Gene expression changes in regulation of lipolysis in adipocytes and AGE-RAGE in diabetic com-plications signaling pathway (GSEA).

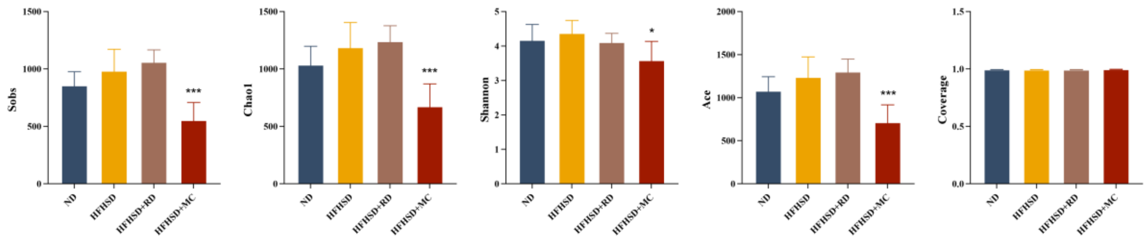

Figure S7: Alpha diversity of gut microbiota in various groups of mice.

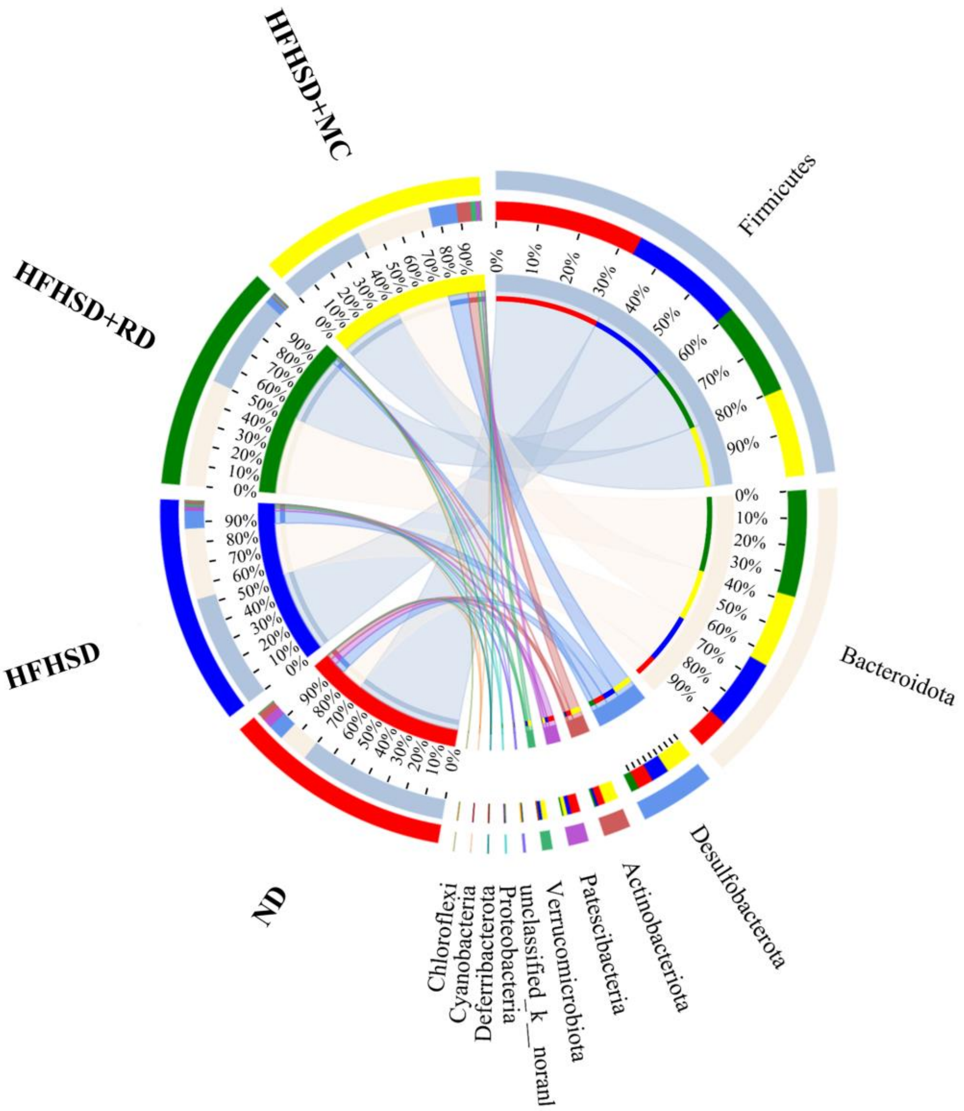

Figure S8: Circos plot of gut microbiota in mice at the phylum level.

Note:
* $p < 0.05$, ** $p < 0.01$, and *** $p < 0.001$ vs. HFHSD group. one-way ANOVA with Tukey's post hoc test

Supplementary Tables

Table S1: PI3K-AKT signaling pathway genes expressions between ND and HFHSD+RD groups.

Symbol	logFC	FDR	ND		HFHSD+RD	
			A1	A9	D2	D10
Pik3r1	0.872091	0.505489	52.11876	80.00097	61.12443	141.3411
Pik3cb	0.495153	0.816115	33.02178	40.23851	31.16267	54.63974
Pik3r6	0.513844	0.89902	6.994439	7.245316	7.844489	8.737237
Akt2	0.943099	0.45505	100.5768	224.6405	234.0731	267.9233
Irs1	0.788853	0.556015	7.605416	16.0059	9.898369	24.10898
Igf1	0.646271	0.725064	58.03018	117.4955	92.70411	130.6163
Slc2a4	1.307523	0.123497	71.43587	137.0394	195.6836	219.4893
Foxo1	0.08354	1	29.17967	35.73065	23.55319	32.96487

Table S2: Expression of selected genes between HFHSD and HFHSD+RD groups.

Symbol	logFC	FDR	HFHSD		HFHSD+RD	
			B1	B3	D2	D10
Cidea	3.341604	1.73×10^{-11}	2.920198	3.718662	48.66589	41.46478
Otop1	4.747068	3.73×10^{-08}	0.052554	0.084043	2.382805	2.681628
Cmklr1	4.570568	5.45×10^{-38}	2.866216	5.149498	125.3139	127.5116
Cebpa	3.892028	4.36×10^{-32}	50.57511	49.1091	952.6121	1076.861
Pparg	4.142995	2.44×10^{-31}	7.779559	15.41245	247.8815	297.108
Adipoq	4.07133	1.30×10^{-30}	300.4137	516.5866	9233.339	8971.886
